# Injury to the Cartilages of the Ribs—Removal of a Portion of Them, and Cure

**Published:** 1842-09

**Authors:** 


*Injury to the Cartilages of the Ribs—Removal of a portion of them, and Cure.*—M. L., aged twenty-eight, was admitted into the University College Hospital, February 21, under the care of Mr. Liston. On the night of the 20th, he took six-penny-worth of laudanum for the purpose of self-destruction, but finding no effect from it, he took a dinner-knife, sharpened the point as well as he could on the hearth-stone, put it to his left side, rested the handle on the bed, and fell upon it. Says he tried to pass the knife between the ribs, but could not. There was some bleeding at first, but it soon ceased, and he tried several times to renew the bleeding, by passing the knife into the wound, and moving it about. He was brought into the hospital at three, P. M.

He is a tall, powerful man, good conformation, sanguine temperament, light complexion, and red hair. Has a wound three-quarters of an inch in length in the left side of the ensiform cartilage, not far from the attachment of the diaphragm. The bleeding had ceased; the wound was dressed with water-dressing. Ordered to have a purgative pill, followed by a dose of castor oil.

Feb. 22. Medicine has not operated; pulse 96, full, and rather inclined to be hard; breathes somewhat quicker than natural; great thirst; tongue covered with a white fur; wound painful, increased by coughing or lying upon his right side; mind much agitated; bowels open at four, P. M.

23. Pulse 104, soft and compressible; complains of pain in the wound on inspiration; less fever.

24. Improving; the wound has begun to suppurate.

26. Attacked in the night with violent pain in the side and dyspnœa; bled to forty ounces with considerable relief.

28. Health much improved; the wound discharges a darkcolored matter, supposed to be the breaking up of a large coagulum. Made an out-patient on the 7th of March.

March 12. Returned to the hospital to-day, complaining that the wound of the side discharged a great deal on walking; walking and other exertion very much affected his breath, and caused a pain in his side, rather lower than the situation of the opening, Dressed with water-dressing, and a broad bandage to be applied round the chest.

14. Discharge continues very profuse; pressure on the parietes of the chest produces no increase of discharge, though it may be seen to ooze out of the wound during a forced inspiration; his health remains good; tongue clean.

16. Mr. Liston passed a probe to the depth of at least two inches into the wound. A collection of matter is supposed to exist somewhere about the attachment of the diaphragm to the cartilages of the last true ribs. Ordered opening medicine.

18. Mr. Liston made a T-shaped incision, passing through the original wound, and on dissecting back the flaps a small opening was seen passing through the cartilages of the ribs where they were united together, to the left of the ensiform cartilage, through which the matter was seen to proceed. A circular portion of two cartilages, nearly the size of a shilling. was removed with a strong blunt-pointed bistoury, half an inch of the point being broken off, and a large quantity of healthy looking matter gushed out. The bleeding was restrained by lint dipped in cold water, and the wound afterwards dressed with water-dressing.

19. No unfavorable symptoms; the wound discharges freely.

20. Complains of pain on inspiration, and uneasiness around the wound. Ordered three grains of calomel and one of opium every three hours.

22. Pain relieved; slight cough; bowels open.

25. Pain entirely gone, the wound contracting and granulating very nicely; health good; appetite voracious; tongue clean. Allowed to walk out every day.

30. Still improving in every respect.

April 8. Discharged cured.—*Phil. Med. Examiner,* from the *London Lancet,* May 14, 1842.

